# Tuina for diabetes with obesity: Protocol for a systematic review and meta-analysis

**DOI:** 10.1097/MD.0000000000023918

**Published:** 2021-01-22

**Authors:** Sihan Peng, Ziyan Xie, Xiyu Zhang, Ya Liu, Xiangeng Zhang, Xiaoli Liang, Hongyan Wang, Chunguang Xie

**Affiliations:** a Hospital of Chengdu University of Traditional Chinese Medicine; b Chengdu University of Traditional Chinese Medicine; c Sichuan Nursing Vocational College; d Sichuan Collaborative Innovation Center for Old-age Care and Elderly Health, Chengdu, Sichuan Province, China.

**Keywords:** diabetes with obesity, meta-analysis, protocol, systematic review, tuina

## Abstract

**Background:**

Obesity is an independent risk factor for the occurrence and development of diabetes. Patients with diabetes combined with obesity will face serious burdens such as increase in insulin resistance and difficulty in blood glucose control. As a safe, effective, economical, and simple intervention, Tuina is more acceptable to the public than drugs. The objective of this systematic evaluation and meta-analysis is to evaluate the efficacy and safety of Tuina for diabetes with obesity.

**Methods:**

We will search the following electronic databases: PubMed, Embase, Cochrane Library, Web of science, Chinese National Knowledge Infrastructure (CNKI), Sino Med, Wanfang, Chinese Clinical Trial Registry System, China Biomedical Literature Database (CBM). The time limit for retrieving studies is from establishment to November 2020 for each database. Randomized controlled clinical trials related to Tuina intervention on diabetes with obesity will be included. Data synthesis, sensitivity analysis, subgroup analysis as well as the assessment of bias risk will be conducted by using Stata V.13.0 and Review manager 5.3 software.

**Results:**

This study will provide a quantitative and standardized evaluation for the efficacy of Tuina therapy on diabetes with obesity.

**Conclusion:**

This systematic review and meta-analysis will provide the high-quality evidence of whether Tuina is an effective intervention for diabetes with obesity.

**Registration number:**

INPLASY2020110106.

## 1 Introduction

Obesity has been confirmed by a large number of studies as a catalyst for type 2 diabetes mellitus (T2DM), and it is also an independent risk factor for the occurrence and development of diabetes.^[1]^ The rise of weight or waist will increase insulin resistance and difficulty in blood glucose control, etc. This means that people with T2DM combined with obesity will face more serious burdens.^[2]^ Obesity is a chronic metabolic disease characterized by visceral and subcutaneous fat accumulation due to the interaction of genetic information and environmental factors.^[3]^ With the changes of human lifestyle and diet structure, the incidence of obesity in the world is increasing year by year. The overweight and obese population in the United States accounted for >60% of the total population in 2012,^[4]^ and obese diabetes accounted for about 60% to 70% of the total diabetes.^[5]^ In addition, obesity is often closely associated with coronary heart disease,^[6]^ hypertension,^[7]^ dyslipidemia,^[8]^ hyperuricemia,^[9]^ fatty liver,^[10]^ and other diseases.

Body mass index (BMI), which combines height and weight to determine obesity, is the most important indicator for the diagnosis and assessment of obesity. WHO defines obesity as the BMI ≥28 kg/m^2^ in Asian people and ≥30 kg/m^2^ in non-Asian people.^[11]^ T2DM was defined as fasting blood glucose (FBG) ≥126.1 mg/dL, HbA1c ≥6.5%, or random blood glucose ≥200.0 mg/dL, excluding other types of diabetes.^[12]^ The management and treatment of diabetes with obesity including lifestyle intervention, medication, and metabolic surgery. Among them, weight management plays an important role in the treatment of diabetes with obesity. The reduce of weight is helpful to decrease their blood sugar level and improve insulin resistance, as well as reduce the application of drugs. The American heart Association and the American obesity Association released “The journal of the American adult obesity and overweight management guide (2013 edition)”^[13]^ in 2013, and the American Diabetes Association (ADA) published “Diabetes medicine diagnosis and treatment standards (2018 edition)”^[14]^ in 2018 have pointed out that weight loss above 5% would benefit in blood sugar control, sustained weight loss of 7%, or higher would have best effective in blood sugar, blood pressure, blood lipid comprehensive treatment.

As a complementary and alternative traditional Chinese medicine (TCM) therapy, Chinese Tuina massage, also called Tuina in China, is an ancient TCM treatment method.^[15]^ It is based on the basic theory of TCM as well as the theory of viscera and meridians, combined with the anatomy of western medicine. Manipulation is applied to the specific part of the body surface to regulate physiological and pathological conditions of the body, so as to dredge the channels and collaterals, regulate Qi and blood, dispel pathogenic factors and strengthen health, and harmonize Yin and Yang. Tuina, including acupuncture, moxibustion, Taichi and Qigong, are representative of non-drug therapies TCM.^[16]^ As a safe, effective, economical, and simple intervention method, Tuina is more acceptable to the public than drugs.^[17]^ The purpose of this study is to evaluate the efficacy and safety of Tuina in the treatment of diabetes with obesity, and to provide evidence for the treatment of diabetes with obesity.

## 2 Methods

### 2.1 Study registration

This protocol has been registered on International Platform of Registered Systematic Review and Meta-Analysis Protocols (INPLASY) with registration number INPLASY2020110106.

### 2.2 The inclusion criteria

#### 2.2.1 Types of studies

We will include the randomized controlled clinical trials and quasi-randomized controlled trials in this study. The studies involving non-randomized controlled trials (RCTs), animal experiments, reviews, and case series will be excluded.

#### 2.2.2 Types of patients

Patients diagnosed with diabetes and obesity (aged ≥18 years) will be included in this study. There is no limitation on the sex, ethnicity, and nation.

#### 2.2.3 Types of interventions

This meta-analysis will include the RCTs of Tuina regardless of the parts, duration, and frequency of Tuina.

#### 2.2.4 Types of comparator(s)/control

Conventional treatment or exercise according to relevant guideline, or other forms of Chinese traditional non-drug intervention such as acupuncture, Qigong, etc. Studies that compared Tuina plus another therapy with the same another therapy alone will be tolerated.

#### 2.2.5 Types of outcomes

The main outcomes of this review are FBG, 2 hour postprandian blood glucose (2hPG), and the weight. The additional outcomes include HbA1c, reduced symptom scores, etc.

### 2.3 Collection and analysis of data

#### 2.3.1 Search strategy

We will search the studies published on PubMed, Embase, Cochrane Library, Web of science, Chinese National Knowledge Infrastructure (CNKI), Sino Med, Wanfang, Chinese Clinical Trial Registry System, China Biomedical Literature Database (CBM), since the inception to November 2020 without language restrictions. The detailed information of electronic search is listed in Table 1.

**Table 1:** Search tactics for the PubMed and web of science.

1 Tuina	7 diabetes
2 Tuina therapy	8 diabetes with obesity
3 Chinese Tuina massage	9 randomized controlled trial
4 traditional Tuina massage	10 randomized trial
5 traditional Chinese therapy	11 controlled clinical trial
6 Acupuncture	12 clinical trial

#### 2.3.2 Data selection

**Figure 1:**
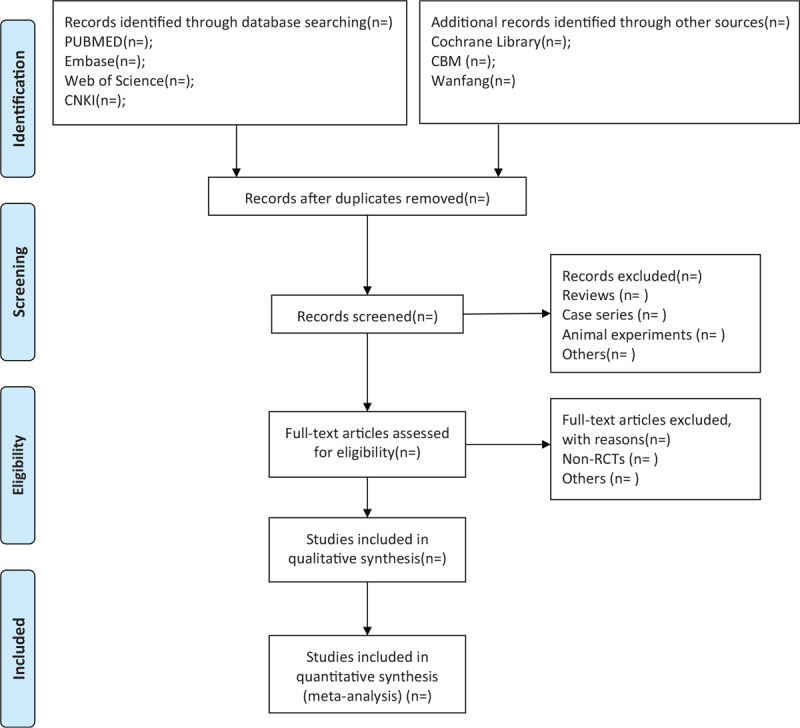
Flow diagram of studies selection process.

Firstly all of the authors will be trained with the Preferred Reporting Items for Systematic Reviews and Meta-Analyses (PRISMA) and Cochrane Handbook for Systematic Reviews of Interventions (CHSRI). The literature will be managed and duplications will be removed by Endnote software (Endnote V.X9). The 2 independent investigators will screen the titles and abstracts of selected studies, and all repetitions and studies not met the inclusion criteria will be excluded. Then the same investigators will review the full texts. Any disagreements will be settled by discussion between the authors. Each process of this study will be performed by using the PRISMA guidelines.^[18]^ In addition, the diagram of this study will be showed in Fig. 1.

#### 2.3.3 Data extraction

According to the eligibility criteria, 2 reviewers will extract data independently from the included studies. The following information will be documented from all the included studies: study characteristics (title, first author, publication year, study design, sample size, setting, randomization methodology, allocation concealment, blinding), participant characteristics (age, sex, etc), intervention details (parts of Tuina, duration of Tuina, etc), outcome indicators for efficacy and safety, follow-up, and others. Any disagreements will be rechecked and resolved by consensus. If the data in the research is unclear or missing, the corresponding author will be contacted for more related information.

#### 2.3.4 Risk of bias assessment

The risk of bias will be assessed independently according to the Cochrane Handbook for Systematic Reviews of Interventions by 2 reviewers. Random sequence generation, allocation concealment, blinding of participants and personnel, the blindness of outcome assessments, incomplete outcome data, selective outcome reporting, and other bias will be evaluated as low risk, high risk, or ambiguous risk in each RCT. The results will be checked repeatedly and the disagreement will be resolved by further discussion of all investigators.

### 2.4 Statistical analysis

#### 2.4.1 Assessment of heterogeneity

The clinical heterogeneity will be evaluated at the beginning. Clinical heterogeneity refers to the variation caused by different patients, interventions, and different end-point indicators of the research. Then statistical heterogeneity will evaluated using the Cochran *Q* and *I*^2^ test if there is no clinical heterogeneity. For the *I*^2^ statistic, *I*^2^ thresholds of <25%, 25% to 49%, 50% to 75%, and >75% to represent low, moderate, high, and very high heterogeneity respectively. The cause of the heterogeneity will be analyzed and a subgroup analysis will be conducted.

#### 2.4.2 Synthesis of data

The Review Manage software will be utilized to analyze all data. We will calculate the risk ratio (RR) for dichotomous with 95% confidence intervals (CIs), and the mean difference (MD) will be included in the meta-analysis for continuous data. While the outcome variables are measured by different scales, standard mean differences (SMD) analysis with 95% CI will also be estimated in the meta-analysis.

#### 2.4.3 Sensitivity analysis

Sensitivity analysis is an important method in order to assess the robustness and reliability of the combined results in meta-analysis. Sensitivity analysis will be required if there are possible low-quality studies after the quality assessment of the included researches. We will observe fluctuation of termination by changing the type of research and reanalysis of the indistinct data.

#### 2.4.4 Subgroup analysis

Subgroup analysis will be performed to evaluate the high heterogeneity of included researches. We will conduct subgroup analysis based on the different acupuncture points for Tuina, duration of Tuina, frequency of Tuina, etc.

#### 2.4.5 Publication bias

If the meta-analysis including >10 trails, a funnel plot will be generated to evaluate potential publication bias. And the symmetrical funnel plot expresses a low risk of publication bias, while the asymmetrical funnel plot indicates high risk.

### 2.5 Quality of evidence

The quality of evidence will be assessed by 2 reviewers independently according to the Grading of Recommendations Assessment, Development and Evaluation (GRADE) system. The quality of evidence is divided into 4 levels (high, moderate, low, and very low), which based on 5 factors (limitation, inaccuracy, inconsistency, indirectness, and publication bias) of GRADE rating standards. The GRADE profiler 3.2 will be employed for analysis.

### 2.6 Ethics and dissemination

Because there are no clinical trial and animal experiment, the ethical approval is not required in this study. Our research results will provide information about the treatment efficacy of Tuina therapy for diabetes with obesity patients. The establishment of this research may be published in peer-reviewed journals.

## 3 Discussion

Tuina is a traditional non-drug therapy in TCM. It has the curative effect of dredging meridians, regulating Qi and blood, dispelling pathogenic factors and strengthening health, and harmonizing Yin and Yang. As a safe, effective, economical and simple intervention method, Tuina is easier to be accepted and adhere to treatment than drugs. However, there has been no systematic meta-analysis of the effect of Tuina on the treatment of diabetes with obesity. Therefore, we intend to conduct a meta-analysis of the effectiveness of Tuina in treating diabetes with obesity, in order to provide high-quality evidence and guidance to clinicians as well as researchers. In addition, we hope to promote further research and development of Tuina.

## Author contributions

**Conceptualization:** Sihan Peng.

**Data collection:** Sihan Peng, Ziyan Xie, Xiyu Zhang.

**Data curation:** Ziyan Xie, Xiyu Zhang, Ya Liu, Hongyan Wang.

**Funding acquisition:** Xiaoli Liang.

**Software:** Ziyan Xie, Xiaoli Liang.

**Statistical analysis:** Ya Liu, Hongyan Wang.

**Supervision:** Xiangeng Zhang.

**Writing – original draft:** Sihan Peng, Xiyu Zhang.

**Writing – review & editing:** Chunguang Xie.
